# Sex differences in metabolic regulation by Gi/o-coupled receptor modulation of exocytosis

**DOI:** 10.3389/fphar.2025.1544456

**Published:** 2025-03-19

**Authors:** Montana Young, Ryan P. Ceddia, Analisa Thompson-Gray, David Reyes, Jackson B. Cassada, Julio E. Ayala, Owen P. McGuinness, Sheila Collins, Heidi E. Hamm

**Affiliations:** ^1^ Department of Pharmacology, School of Medicine, Vanderbilt University, Nashville, TN, United States; ^2^ Division of Cardiovascular Medicine, Department of Medicine, Vanderbilt University Medical Center, Nashville, TN, United States; ^3^ Department of Molecular Physiology and Biophysics, School of Medicine, Vanderbilt University, Nashville, IN, United States

**Keywords:** g protein coupled receptor (GPCR), sex hormone, metabolism, norepinephrene, diet induced obesity (DIO), exocytosis, SNARE (soluble N-ethylmaleimide-sensitive fusion protein attachment protein receptor), g protein beta gamma

## Abstract

**Background:**

Presynaptic G_i/o_ coupled GPCRs can act as negative feedback regulators of neurotransmitter release via Gβγ effector modulation through two mechanisms: decreased calcium influx and direct inhibition of membrane fusion by soluble N-ethylmaleimide—sensitive factor attachment protein (SNAP) receptor (SNARE). Previously, we discovered that truncation of the last three C-terminal amino acids of SNAP25 (SNAP25Δ3) prevents Gβγ-SNARE interaction, effectively removing the braking mechanism on neurotransmitter release. We have demonstrated enhanced metabolic protection in male SNAP25^Δ3/Δ3^ mice housed at room temperature (22°C), including increased adipose tissue beiging and glucose uptake and enhanced insulin sensitivity, rendering them resistant to diet-induced obesity (DIO). When male SNAP25^Δ3/Δ3^ mice were housed at thermoneutrality (30°C), all metabolic protection was abolished, suggesting sympathetic tone is important for the phenotypes.

**Methods:**

We housed male and female mice at either standard room temperature (21°C) or at thermoneutrality (30°C) and fed them a high fat diet (HFD) for 8 weeks. Glucose tolerance tests were performed before and after the 8 weeks of HFD along with body composition analyses. Organs were then dissected for mass analysis as well as immunohistochemistry. Additionally, we ovariectomized female mice to investigate the role of sex hormones in our phenotypes. Finally, we housed mice in Sable Promethion chambers at various environmental temperatures to investigate the effect of environmental temperature on basal metabolic rates.

**Results:**

We found SNAP25^Δ3/Δ3^ female mice exhibited the same metabolic protection at RT (22°C) and displayed enhanced metabolic protection from DIO compared to standard chow just as males did. However, female SNAP25^Δ3/Δ3^ mice display persistent metabolic protection even when housed at thermoneutrality. In this study, we investigate the mechanisms behind this sex dependent persistent phenotype. Thermoneutral set point did not differ between sexes nor genotype, suggesting that metabolic protection is not due to a difference in hypothalamic temperature regulation. Metabolic protection in SNAP25^Δ3/Δ3^ persisted in ovariectomized mice despite increased weight gain compared to mice receiving sham operations.

**Conclusion:**

This study has identified that there is not a sex-dependent difference for thermoneutral set point in mice. Additionally, there is a sex hormone independent mechanism driving the persistent metabolic protection of female SNAP25^Δ3/Δ3^ mice housed in thermoneutrality.

## Introduction

Metabolic diseases have an enormous impact on the healthcare system and are only expected to increase in prevalence over the next 50 years (Saklayen, 2018). Therapeutics targeting G-protein coupled receptors (GPCRs) have shown promise in a wide variety of disease conditions, including aiding metabolic dysfunction, but these compounds often cause many side effects through Gα subunit signaling (Lau et al., 2015; Hall et al., 2018; Bucheit et al., 2020). Though systemic effects also occur when Gβγ signaling effectors are modulated, many roles of Gβγ have either been ignored or overlooked (Smrcka, 2008). Our previous work on the regulation of exocytosis in excitable cells has shown that Gβγ binds to the neuronal SNARE complex (made up of SNAP25, syntaxin 1 and synaptobrevin-2/VAMP2), inhibiting exocytosis via the 5HT_1b_ receptor (Blackmer et al., 1979; Photowala et al., 2006). We found that removing the last three amino acids from the C-terminal end of SNAP25 (SNAP25Δ3) effectively disables the ability of Gβγ to inhibit calcium-synaptotagmin-stimulated neurotransmitter exocytosis (Zurawski et al., 2016). Since deletion of these amino acids only affects the SNAP25 effector, all other aspects of G_i/o_ GPCR signaling should be intact. This specifically includes the functionality of Gα_i/o_ modulation of adenylyl cyclase, Gβγ inhibition of calcium channels, and the other downstream effects of Gβγ-mediated signaling.

To investigate the physiological effects of the SNAP25Δ3 mutation *in vivo* we generated a mouse that expresses SNAP25Δ3 globally (Zurawski et al., 2019). This SNAP25^Δ3/Δ3^ mouse highlighted the importance of the Gβγ-SNARE interaction in pathways such as stress response, spatial memory learning, and pain signaling (Zurawski et al., 2019; Thompson Gray et al., 2020). These behavioral and physiological phenotypes also displayed sex-dependent differential magnitudes of the effect (Thompson Gray et al., 2020).

Significant metabolic phenotypes were observed in the SNAP25^Δ3/Δ3^ mouse model, notably male mice displayed protection from diet-induced obesity (DIO) and improved glucose handling (Ceddia et al., 2023). These male mice also displayed increased inguinal white adipose tissue (iWAT) beiging and a concomitant elevation of norepinephrine in white adipose tissue when housed at room temperature (22°C) (Ceddia et al., 2023). Interestingly, this metabolic protection phenotype was abolished when the male mice were housed at thermoneutrality (30°C) (Ceddia et al., 2023). Sympathetic neurons from iWAT of SNAP25^Δ3/Δ3^ mice release significantly more norepinephrine in response to electrical stimulation than wild-type mice, consistent with ablation of Gβγ-inhibition of neurotransmitter exocytosis. Collectively, the increased norepinephrine release from adipose tissue sympathetic neurons paired with temperature-dependent elevation of adipose tissue norepinephrine levels and adipocyte browning leads us to hypothesize increased sympathetic tone due to the loss of Gβγ-SNARE regulation of norepinephrine exocytosis is responsible for the metabolic phenotypes seen in these mice. Increased adipose tissue browning is known to be an effect downstream of the three adrenergic receptors and considering that mice experience mild thermal stress at room temperature (22°C), norepinephrine-mediated temperature regulation is likely the mechanism behind these metabolic phenotypes (Ceddia et al., 2023; Cannon and Nedergaard, 2004; Ceddia and Collins, 2020).

The SNAP25^Δ3/Δ3^ mouse model has consistently shown sexually dimorphic phenotypes (Thompson Gray et al., 2020). Here, we examine the metabolic phenotypes of female SNAP25^Δ3/Δ3^ mice and how they differ from male SNAP25^Δ3/Δ3^ mice. We also describe the metabolic effects of environmental temperature differences between the sexes of mice. Finally, since our results indicate that female SNAP25^Δ3/Δ3^ mice display a sex-dependent metabolic phenotype, we investigated the role of ovariectomy in sexually dimorphic metabolic phenotypes.

## Results

### Protection from diet-induced obesity at standard room temperature housing (22°C) in male and female SNAP25^Δ3/Δ3^ mice

**FIGURE 1:**
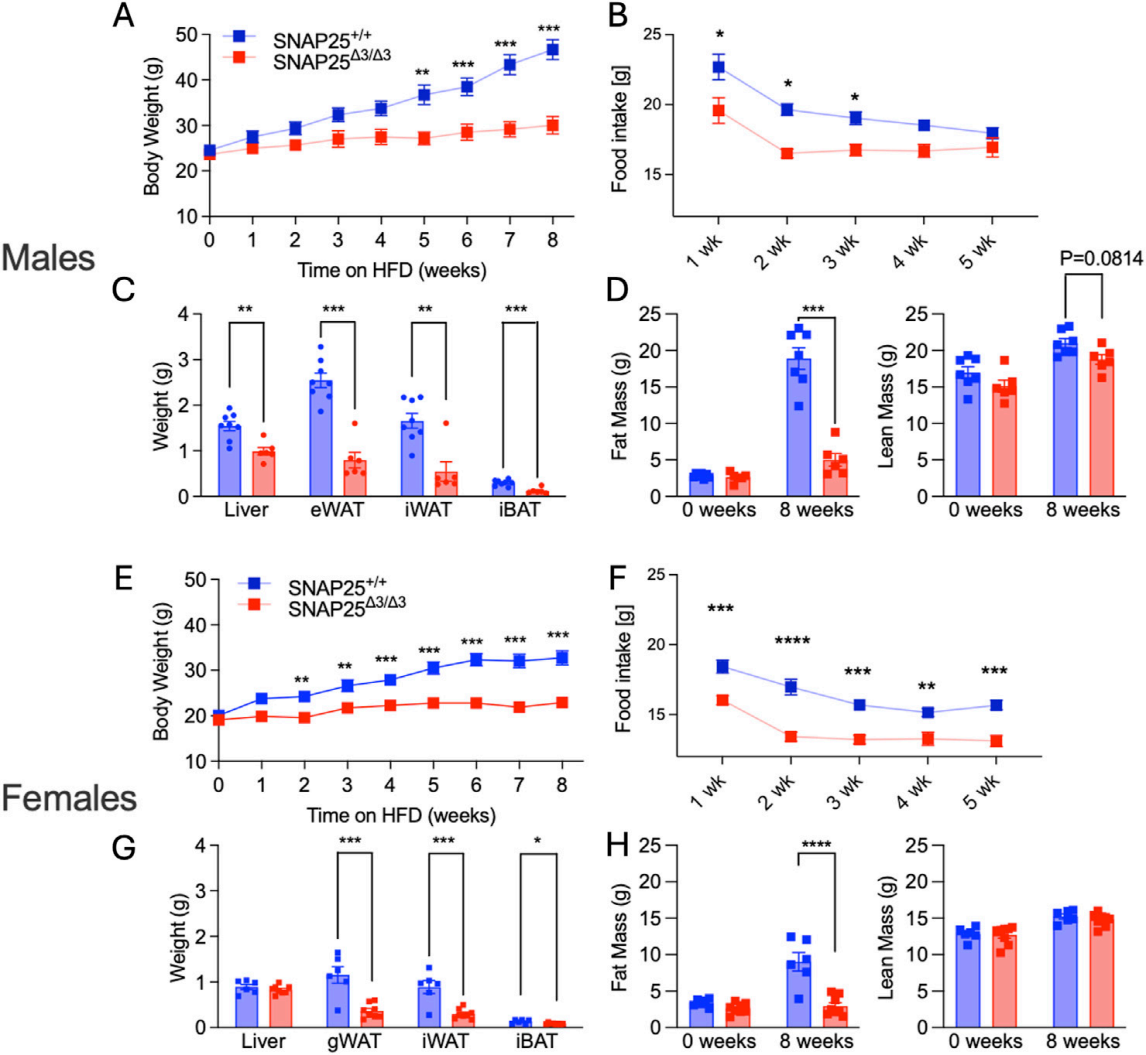
Room Temperature HFD Study. Males **(A-D)**; females **(E-H)**. Body weight of SNAP25^+/+^ and SNAP25^Δ3/Δ3^ male **(A)** and female **(E)** mice at 22°C on HFD analyzed by two-way ANOVA with Bonferroni’s post-hoc comparison. Male n = 23 SNAP25^+/+^ mice; n = 18 SNAP25^Δ3/Δ3^; female n = 13 SNAP25^+/+^ mice; n = 14 SNAP25^Δ3/Δ3^. (**=P ≤ 0.01; ***=P ≤ 0.001; ****=P ≤ 0.0001). Genotype effect: male = P ≤ 0.003; female = P ≤ 0.0001. Food intake from the chow-adapted male **(B)** and female **(F)** SNAP25^+/+^ and SNAP25^Δ3/Δ3^ mice given ad libitum access to HFD. Both male and female n = 5 SNAP25^+/+^ mice; n = 6 SNAP25^Δ3/Δ3^ mice. (*=P < 0.05). Values are the mean ± SEM. Analyses were performed using 2-way ANOVA, and post hoc analyses were performed using Bonferroni’s multiple-comparison test for the SNAP25 genotype only. Genotype effect: male = P ≤ 0.001; female = P ≤ 0.0001. Male **(C)** and female **(G)** organ weights after 8 weeks of HFD at 22°C Male n = 8 SNAP25^Δ3/Δ3^ mice; n = 6 SNAP25^Δ3/Δ3^ Female n = 6 Snap25^+/+^ mice; n = 8 SNAP25^Δ3/Δ3^ (*=P≤0.001). Analyses performed by multiple unpaired T-Tests. Fat mass at week 0 (left) and after 8 weeks HFD (right) Male **(D)** n = 8 SNAP25^Δ3/Δ3^ mice; n = 6 SNAP25^Δ3/Δ3^; Female **(H)** n = 11 SNAP25^+/+^ mice; n = 12 SNAP25^Δ3/Δ3^ (***=P ≤ 0.001; ****=P ≤ 0.0001). Male Data adapted from Ceddia et al., 2023.

Body weights and food intake were monitored in male and female SNAP25^Δ3/Δ3^ and wild-type littermates for 8 weeks at room temperature (22°C) while fed a high-fat diet. Both male and female SNAP25^Δ3/Δ3^ mice displayed protection from DIO compared to their SNAP25^+/+^ littermates, with female SNAP25^Δ3/Δ3^ mice showing a significant difference in weight between genotypes beginning in week two and male SNAP25^Δ3/Δ3^ mice at week 5 (Figures 1A, E). Food intake was significantly decreased only during the first 3 weeks of HFD feeding in male SNAP25^Δ3/Δ3^ mice. This was consistent with a blunting of the initial hyperphagic response when given the highly palatable HFD. Conversely, in female SNAP25^Δ3/Δ3^ mice, food intake was reduced during all 5 weeks of HFD feeding (Figures 1B, F). Following 8 weeks of HFD feeding the tissue weights of gonadal white adipose tissue (gWAT) and iWAT were decreased in both male and female SNAP25^Δ3/Δ3^ mice (Figures 1C, G). Histological analyses of iWAT, interscapular brown adipose tissue (iBAT), and gWAT showed smaller and more densely packed adipocytes in all fat pads from both male and female SNAP25^Δ3/Δ3^ animals compared to their SNAP25^+/+^ littermates (Supplementary Figure S1). As previously observed in Ceddia et al., 2023, SNAP25^Δ3/Δ3^ males and females, display increased expression of uncoupling protein 1 (UCP1) in their iWAT consistent with the adipose tissue beiging as classically seen in cold exposure (Supplementary Figure S1; Supplementary Figure S7). Body composition analysis on these mice demonstrates that there is a significant decrease in body fat in both male and female SNAP25^Δ3/Δ3^ mice compared to their SNAP25^+/+^ littermates after 8 weeks of *ad libitum* access to HFD (Figures 1D, H). It is important to note that the lean mass between genotypes did not differ suggesting that the increase in body weight observed in the SNAP25^+/+^ mice is due to increased adiposity, not overall mass gain (Figures 1D, H). Circulating leptin levels from these mice were significantly lower in both male and female SNAP25^Δ3/Δ3^ mice compared to their SNAP25^+/+^ littermates, consistent with the fact that there is reduced adipose mass (Supplementary Figure S6). From these results we can conclude that female SNAP25^Δ3/Δ3^ mice display a similar response to male SNAP25^Δ3/Δ3^ mice regarding mild thermal stress leading to increased norepinephrine release and adipose tissue beiging. We can also conclude that these effects are not driven by increased thermogenic drive from enhanced leptin signaling.

### Protection from diet-induced obesity at thermoneutrality (30°C) persists only in female SNAP25^Δ3/Δ3^ mice

**FIGURE 2:**
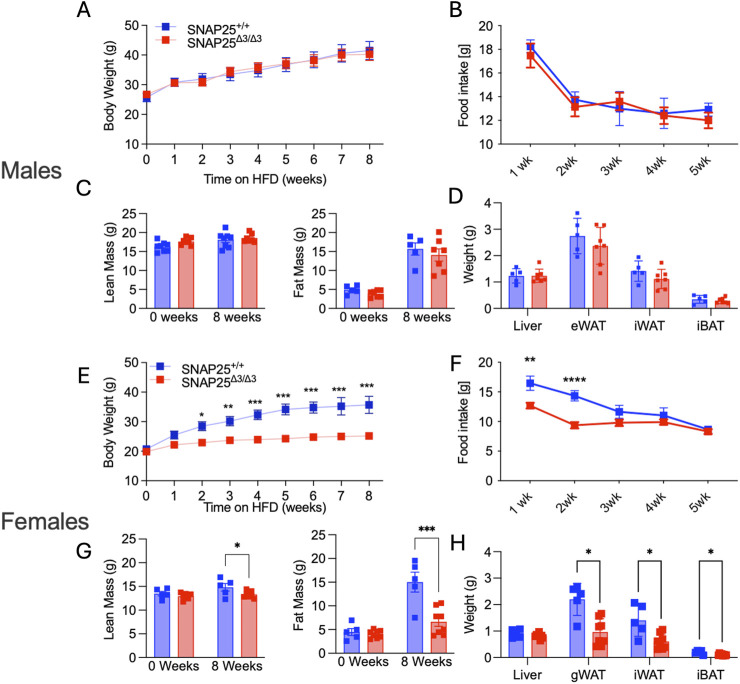
Thermoneutral HFD Study. Males **(A-D)**; Females **(E-H)**. Body weight of SNAP25^+/+^ and SNAP25^Δ3/Δ3^ male **(A)** and female **(E)** mice at 29°C on HFD. Male (A) n = 8 SNAP25^+/+^ mice; n = 7 SNAP25^Δ3/Δ3^; female **(E)** n = 5 SNAP25^+/+^ mice; n = 8 SNAP25^Δ3/Δ3^. (*=P≤0.05; **=P≤0.01; ***=P≤0.001; ****=P≤0.0001). Analyses were performed using 2-way ANOVA, and post hoc analyses were performed using Bonferroni’s multiple-comparison test for the SNAP25^Δ3/Δ3^ genotype only. Genotype effect: male = P ≤ 0.99; female = P ≤ 0.001. Male **(B)** and female **(F)** food intake from male mice given ad libitum access to HFD. Male n = 8 SNAP25^+/+^ mice; n = 7 SNAP25^Δ3/Δ3^; female n = 5 SNAP25^+/+^ mice; n = 8 SNAP25^Δ3/Δ3^. (**=P ≤ 0.01; ****= P≤0.0001) Values are the mean ± SEM. Analyses were performed using 2-way ANOVA, and post hoc analyses were performed using Bonferroni’s multiple-comparison test for the SNAP25^Δ3/Δ3^ genotype only. Genotype effect: male = P ≤ 0.657; female = P ≤ 0.0017. Fat Mass at week 0 (left) and after 8 weeks HFD (right) male **(C)** n = 5 SNAP25^+/+^ mice; n = 7 SNAP25^Δ3/Δ3^; female **(G)** n = 5 SNAP25^+/+^ mice; n = 8 SNAP25^Δ3/Δ3^ (***=P ≤ 0.001; ****=P≤0.0001). Organ weights after 8 weeks of HFD male **(D)** n = 5 SNAP25^+/+^ mice; n = 7 SNAP25^Δ3/Δ3^ female **(H)** n = 5 SNAP25^+/+^ mice; n = 8 SNAP25^Δ3/Δ3^ (*=P ≤ 0.01). Analyses performed by multiple unpaired T-Tests. Male Data adapted from Ceddia et al., 2023.

A complete loss of metabolic protection in SNAP25^Δ3/Δ3^ male mice when housed at thermoneutral conditions (30°C) was observed in our prior studies (Ceddia et al., 2023). Therefore, we placed female SNAP25^Δ3/Δ3^ and SNAP25^+/+^ littermates in 30°C housing with *ad libitum* access to HFD for 8 weeks. Unlike male mice, metabolic protection persisted in female SNAP25^Δ3/Δ3^ mice, which continued to display significantly lower body weights from week two through eight in the same thermoneutral conditions (Figures 2A, E). Body composition also reflects a similar trend to what was seen in the previous 22°C study, where SNAP25^Δ3/Δ3^ female mice had significantly less fat mass compared to SNAP25^+/+^ littermates (Figures 2C, G). While there was a small difference in lean mass between the two genotypes of female mice, this difference cannot explain the difference in total body weight (Figures 2A, E). Unlike male mice, female SNAP25^Δ3/Δ3^ mice exhibited significantly decreased food intake during the first 2 weeks, suggesting a blunted hyperphagic response to the initial HFD exposure (Figures 2B, F). Organ weight at the end of the 8 weeks reflects what was observed in body composition analysis as there are significant weight differences between female SNAP25^Δ3/Δ3^ and SNAP25^+/+^ littermates in gWAT, iWAT, and iBAT mass (Figures 2D, H). Histology also demonstrates that adipocyte morphology of male mice does not differ between genotypes. In contrast, female SNAP25^Δ3/Δ3^ adipocytes are persistently smaller and more densely packed compared to their SNAP25^+/+^ littermates (Supplementary Figure S3). Interestingly, UCP1 expression is no longer expressed at a higher rate in female SNAP25^Δ3/Δ3^ mice housed at thermoneutrality. To further investigate how female mice are responding to HFD in thermoneutrality compared to males we conducted a glucose tolerance test (GTT) by injecting a bolus of glucose intraperitoneally with both male and female SNAP25^Δ3/Δ3^ and SNAP25^+/+^ littermates. As seen in Supplementary Figure S2, male SNAP25^Δ3/Δ3^ and SNAP25^+/+^ littermates did not show any notable difference in glucose handling. Circulating insulin during a GTT also did not differ significantly between male SNAP25^Δ3/Δ3^ and SNAP25^+/+^ littermates. However, female SNAP25^Δ3/Δ3^ mice had significantly less circulating insulin levels at the 60-min timepoint with comparable blood glucose levels between SNAP25^Δ3/Δ3^ and SNAP25^+/+^ littermates demonstrating enhanced glucose handling. These data suggest a secondary mechanism by which female SNAP25^Δ3/Δ3^ mice respond to thermoneutral housing conditions on HFD, resulting in metabolic protection.

### Thermoneutral set point is unaltered in female SNAP25^Δ3/Δ3^ mice

**FIGURE 3:**
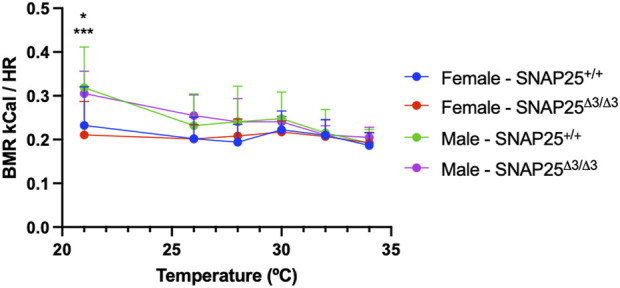
BMR and Environmental Temperature Study. Basal Metabolic Rate (BMR) x Temperature (°C) with male and female mice fed chow diet in the Sable Promethion System. Male n = 6 SNAP25^+/+^ mice; n = 6 SNAP25^Δ3/Δ3^ mice. Female n =12 Snap25^+/+^ mice; n = 12 SNAP25^Δ3/Δ3^ mice. Values are the mean ± SEM. Analyzed by two-way ANOVA with Tukey post-hoc analysis. Gender (column) effect: P = 0.0001. At 21°C: WT male x female = P<0.05; SNAP25Δ3 male x female P<0.001. (*) = P < 0.05 (***) = P < 0.001

Considering the significantly different metabolic phenotypes observed between male and female mice in mild thermal stress and thermoneutrality, we hypothesized that a difference in thermoneutral set point between male and female mice could explain this. A difference in thermoneutral set point would mean that, in our prior studies housing mice at ∼30°C, these conditions were not thermoneutral for the female mice only, and these female mice would still be experiencing a mild cold stress. To test this, we housed male and female SNAP25^Δ3/Δ3^ and SNAP25^+/+^ littermates in a Sable Promethion, which allowed us to monitor food intake, activity levels, respiratory exchange ratios (RER), and basal metabolic rates (BMR) while manipulating temperature. We began by housing the mice at 21°C for a 48 h acclimation period, then increased the environmental temperature to 26°C for 24h, and subsequently raised the temperature 2°C every 24 h until we reached 32°C. At this point, we waited 24 h and then reduced the temperature back to 21°C. Monitoring BMR throughout this temperature ramp protocol, we observed that while female mice did have a lower BMR compared to male mice in both SNAP25^Δ3/Δ3^ and SNAP25^+/+^ animals at 21°C, this difference was abolished as temperature increased, suggesting there was not a difference in the thermoneutral set point between sex nor genotype (Figure 3). As expected, there was not a significant difference in respiratory exchange ratio (RER) or activity between these groups (data not shown). Based on the data obtained with the Sable Promethion, we can conclude that from a metabolic perspective there does not appear to be a different thermoneutral set point between sex or genotype within our mouse population.

### Protection from diet-induced obesity at thermoneutrality (30°C) persists in SNAP25^Δ3/Δ3^ mice after ovariectomy

**FIGURE 4:**
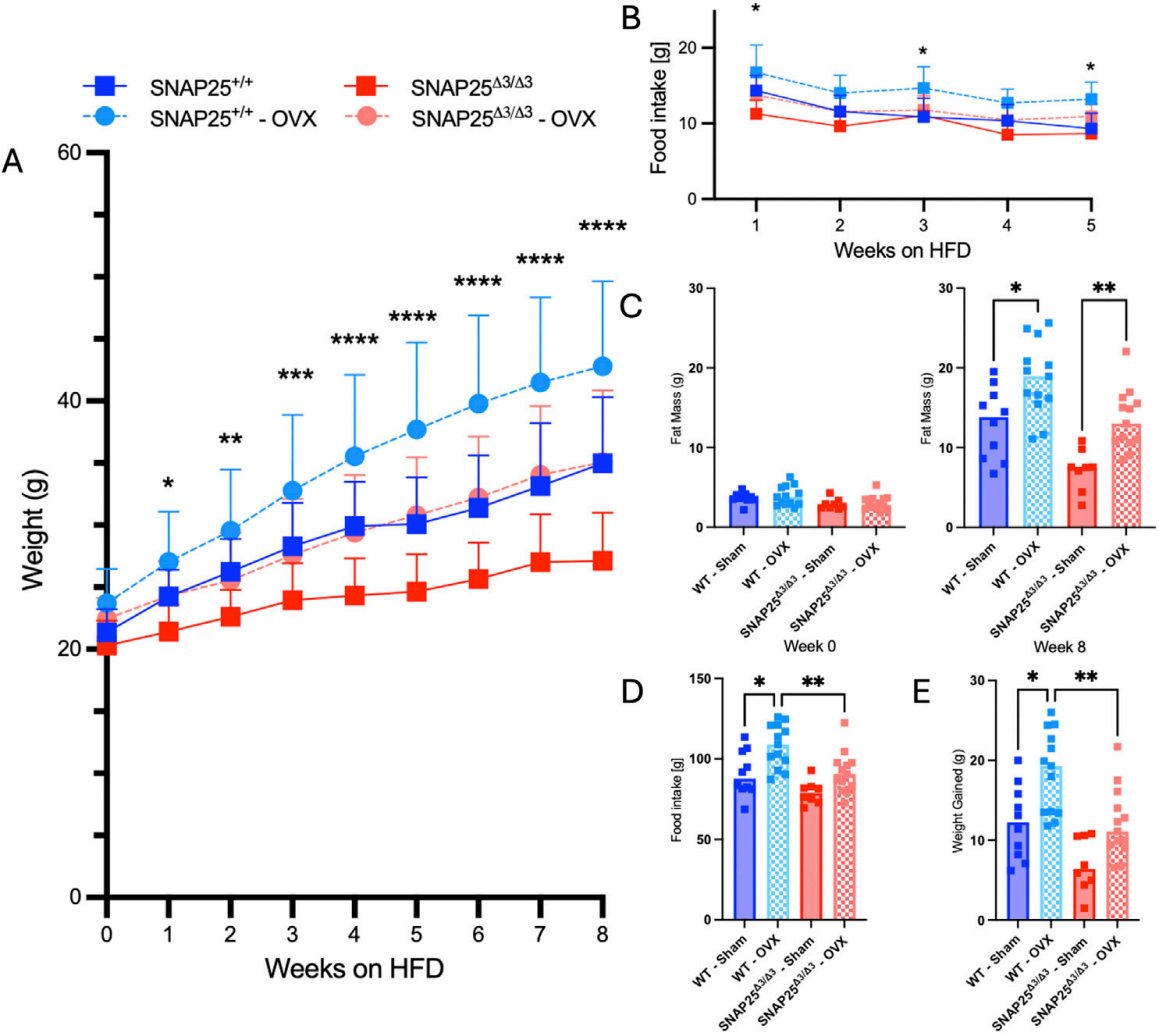
Thermoneutral HFD Study With Ovariectomized And Sham SNAP25^+/+^ And SNAP25^Δ3/Δ3^ Mice. **(A)** Body weight of SNAP25^+/+^ and SNAP25^Δ3/Δ3^ OVX and Sham female mice at 29°C on HFD analyzed by two-way ANOVA with Šídák post-hoc comparison. n = 10 SNAP25^+/+^ sham mice; n = 13 SNAP25^+/+^ OVX mice, n = 10 SNAP25^Δ3/Δ3^ sham mice, n = 13 SNAP25^Δ3/Δ3^ OVX mice (**=P≤0.01; ***=P≤0.001; ****=P≤0.0001). **(B)** Food intake from male and female SNAP25^+/+^ and SNAP25^Δ3/Δ3^ mice given ad libitum access to HFD. n = 10 SNAP25^+/+^ sham mice; n = 13 SNAP25^+/+^ OVX mice, n = 10 SNAP25^Δ3/Δ3^ sham mice, n = 13 SNAP25^Δ3/Δ3^ OVX mice. *=P < 0.05. Values are the mean ± SEM. Analyses were performed using 2-way ANOVA, and post hoc analyses were performed using Tukey’s multiple-comparison test for the SNAP25^Δ3/Δ3^ genotype only. Genotype effect = P < 0.001. **(C)** Fat mass (P < 0.001, genotype; P < 0.001, genotype × time interaction) and lean mass (ns) at the beginning and end of the HFD-feeding period. n = 10 SNAP25^+/+^ sham mice; n = 13 SNAP25^+/+^ OVX mice, n = 10 SNAP25^Δ3/Δ3^ sham mice, n = 13 SNAP25^Δ3/Δ3^ OVX mice. * = P < 0.05; ** = P < 0.01. **(D)** Cumulative food intake over the course of the 8-week HFD study. n = 10 SNAP25^+/+^ sham mice; n = 13 SNAP25^+/+^ OVX mice, n = 10 SNAP25^Δ3/Δ3^ sham mice, n = 13 SNAP25^Δ3/Δ3^ OVX mice. Analyzed by one way ANOVA with Tukey’s post-hoc analysis. * = P < 0.05; ** = P < 0.01. **(E)** Cumulative weight gain over the course of the 8-week HFD study. n = 10 SNAP25^+/+^ sham mice; n = 13 SNAP25^+/+^ OVX mice, n = 10 SNAP25^Δ3/Δ3^ sham mice, n = 13 SNAP25^Δ3/Δ3^ OVX mice. Values are the mean ± SEM. Analyzed by one way ANOVA with Tukey’s post-hoc analysis. * = P < 0.05; ** = P < 0.01.

**FIGURE 5:**
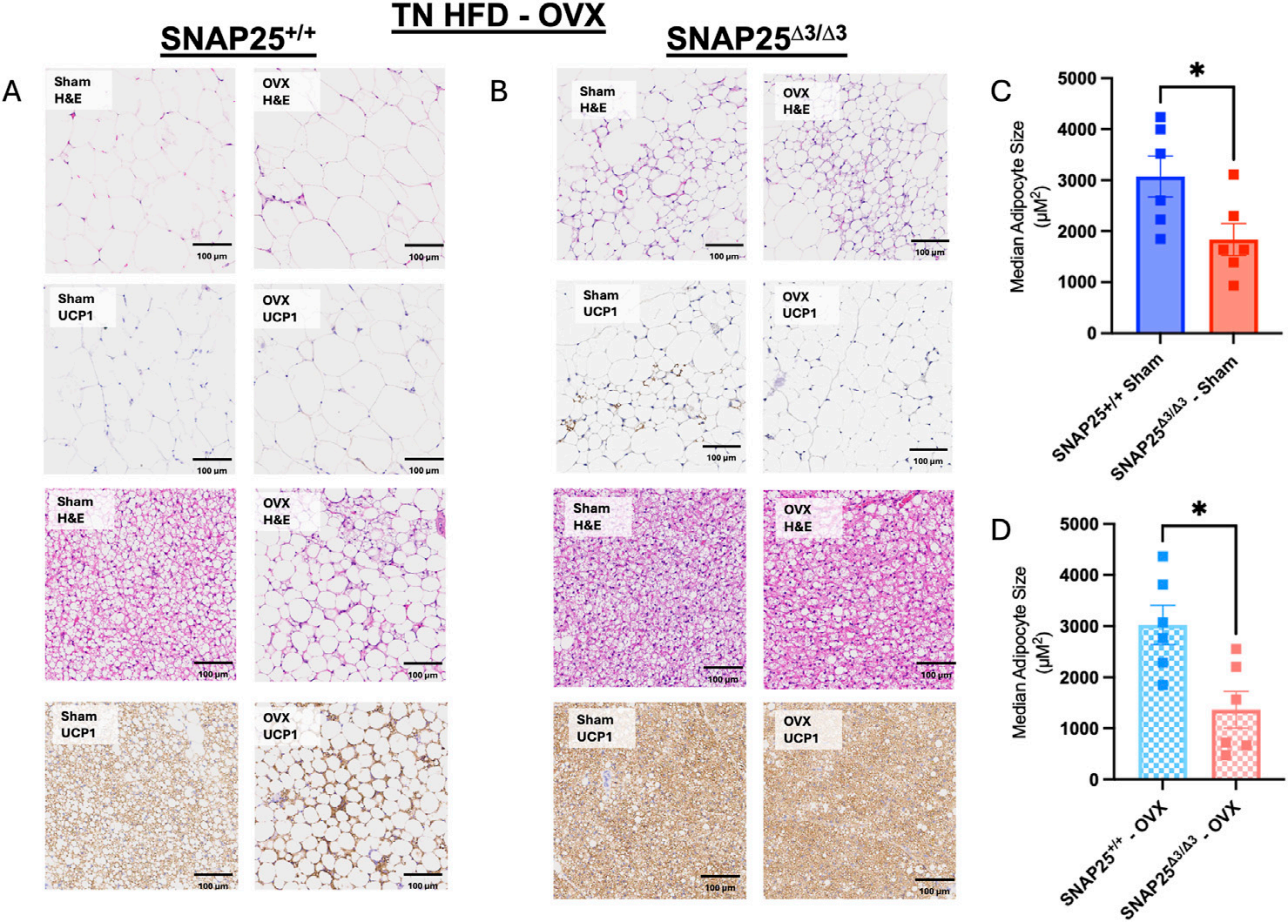
Histology From Thermoneutral HFD Study With Ovariectomized And Sham SNAP25^+/+^ And SNAP25^Δ3/Δ3^ Mice. **(A, B)** Representative H&E and UCP1 of gonadal adipose tissue (top four) and iBAT (bottom four) of OVX and Sham female SNAP25^+/+^ and SNAP25^Δ3/Δ3^ mice fed HFD for 8 weeks at 30°C. Scale bars: 100 μm. Images are a representative sample from 3–4 mice from each group. **(C)** Quantification of hematoxylin and eosin stained iWAT of Sham SNAP25^+/+^ and SNAP25^Δ3/Δ3^ mice with the median cell size for 6 mice from each group plotted. **(D)** Quantification of hematoxylin and eosin stained iWAT of OVX SNAP25^+/+^ and SNAP25^Δ3/Δ3^ mice with the median cell size for 6 mice from each group plotted. Values are the mean ± SEM. Analyzed by unpaired 2-tailed student’s T-Test. (* = P < 0.05).

Considering we continued to observe a female-specific persistence of metabolic protection on HFD at thermoneutrality and there was not a difference in thermoneutral set point in female mice, we hypothesized that there might be a sex hormone dependent effect on the metabolic phenotype of our SNAP25^Δ3/Δ3^ mice. To investigate the potential role of sex hormones in our mice, we repeated the HFD metabolic experiment at thermoneutrality with SNAP25^Δ3/Δ3^ and SNAP25^+/+^ littermates that either received an ovariectomy (OVX), to remove the influence of sex hormones (estrogen, testosterone, and their derivatives such as progesterone) from their physiology, or underwent a sham OVX to account for stress from the OVX procedure (Rowe et al., 2023). Interestingly, throughout the 8 weeks of *ad libitum* access to HFD, the OVX procedure increased HFD-induced weight gain a similar amount in both genotypes (Figure 4A). However, metabolic protection was still observed in the SNAP25^Δ3/Δ3^ mice when compared with SNAP25^+/+^ mice receiving the same procedure. Food intake followed a similar trend to body weight measurements as the OVX increased food intake to a similar extent irrespective of genotype (Figure 4B). This is reflected in the two-way ANOVA in Figure 4A as SNAP25^Δ3/Δ3^ OVX and SNAP25^+/+^ OVX have significantly different bodyweights from week 2 onward as well as SNAP25^Δ3/Δ3^ sham and SNAP25^+/+^ sham displaying significantly different bodyweights from week 6 onward. Figure 4B reveals significantly different food intakes at weeks 1, 3, and 5. It is important to note that the SNAP25^Δ3/Δ3^ phenotype still led to a decreased body weight and food intake regardless of the procedure. Body composition reflects a similar trend. OVX groups had higher body fat than sham groups in both genotypes, but the SNAP25^Δ3/Δ3^ sham and OVX mice had lower amounts of body fat than their SNAP25^+/+^ sham and OVX littermates (Figure 4C). Lean mass did not differ between any of the groups suggesting that the SNAP25^Δ3/Δ3^ genotype and the OVX and sham procedures are specifically affecting adiposity in these mice (Figure 4C). Organ weights also follow the body weight and body composition trend where OVX mice have larger gWAT and iWAT deposits but SNAP25^Δ3/Δ3^ have smaller deposits than SNAP25^+/+^ littermates (Supplementary Figure S5). As seen previously, there were no differences between groups in liver or iBAT mass (Supplementary Figure S5). However, when observing cumulative food intake and cumulative weight gain, SNAP25^Δ3/Δ3^ sham mice have significantly lower food intake and weight gain compared to SNAP25^+/+^ sham mice (Figures 4D, E). Similarly, SNAP25^Δ3/Δ3^ OVX mice have significantly lower food intake and weight gain compared to SNAP25^+/+^ OVX mice. Taken together, these observations reveal that the SNAP25^Δ3/Δ3^ have metabolic protection with or without the presence of sex hormones. Histology revealed some interesting differences. The SNAP25^+/+^ OVX vs sham adipose tissues displays large adipocytes, however, SNAP25^Δ3/Δ3^ adipocytes continued to be smaller and more densely packed compared to SNAP25^+/+^ littermates regardless of surgical procedure (Figure 5). These data suggests that OVX leads to increased food intake and increased overall adipose tissue mass, but the SNAP25^Δ3/Δ3^ genotype still causes adipose tissue remodeling regardless of the presence of ovaries. Similar to the previous thermoneutral housing study with SNAP25^Δ3/Δ3^ female mice, UCP1 expression was very low such that from the iWAT immunohistochemistry slides of sham and OVX mice of both genotypes we cannot conclude that UCP1 expression is elevated. Collectively, these results suggest that sex hormones and NE act on the adipose tissue through separate mechanisms. When observing the response of each group to an intraperitoneal GTT after 8 weeks of HFD at thermoneutrality, the trends of enhanced glucose handling in SNAP25^Δ3/Δ3^ mice return. However, it appears that an OVX negatively impacts glucose tolerance in both SNAP25^Δ3/Δ3^ and SNAP25^+/+^ genotypes indiscriminately, further supporting the claim that enhanced neurotransmitter release and sex hormones are acting through different mechanisms (Supplementary Figure S4). Female SNAP25^Δ3/Δ3^ mice had lower circulating insulin levels in response to a glucose bolus during an intraperitoneal GTT with comparable blood glucose levels between SNAP25^Δ3/Δ3^ and SNAP25^+/+^ littermates demonstrating enhanced glucose handling. OVX caused a slight increase of circulating insulin in both groups regardless of genotype, but the decreased circulating insulin levels persisted in the SNAP25^Δ3/Δ3^ OVX mice. This claim is also supported by the significantly different genotype effect observed when comparing female SNAP25^Δ3/Δ3^ and SNAP25^+/+^ irrespective of OVX for blood glucose and insulin levels during a GTT after 8 weeks of HFD at thermoneutrality. Overall, it seems that the SNAP25^Δ3/Δ3^ metabolic protection is intact regardless of sex hormones; however, it does appear that loss of the estrous cycle through OVX results in evident metabolic disruption.

## Discussion

We began studying the metabolic effects of loss of Gβγ-SNARE inhibition in SNAP25^Δ3/Δ3^ mice, which displayed significant protection from DIO, improved glucose handling, and smaller adipocytes with increased UCP1 expression under mild cold stress that was abolished in thermoneutral conditions. We observed circulating leptin levels were lower in both male and female SNAP25^Δ3/Δ3^ compared to their SNAP25^+/+^ littermates, which is reflective of the reduced adipose tissue mass. Leptin is known to drive thermogenesis, as it has been demonstrated that higher plasma leptin levels correspond with increased body temperature via iBAT thermogenesis (Münzberg and Morrison, 2015; Morrison et al., 2008). Considering male and female SNAP^Δ3/Δ3^ mice had lower amounts of circulating leptin compared to their SNAP25^+/+^ littermates, we can conclude leptin is not driving this phenotype. The cold-stimulated adipose tissue remodeling and beiging in both genotypes provided a clue that this was a NE-mediated mechanism, which was confirmed using live cell fluorescence imaging with the norepinephrine sensor GRAB_NE_ (Ceddia et al., 2023). These results showed an increase in released NE in the adipose tissue of male SNAP25^Δ3/Δ3^ mice (Ceddia et al., 2023). Investigating the phenotypic response of female SNAP25^Δ3/Δ3^ mice resulted in a similar metabolic effect but with a surprising sex difference. Similarly to the male SNAP25^Δ3/Δ3^ mice, female SNAP25^Δ3/Δ3^ mice exhibited metabolic phenotypes consistent with increased NE release in the adipose tissue including protection from DIO, improved glucose handling, and smaller adipocytes with increased UCP1 expression at room temperature. However, this metabolic protection was persistent at thermoneutrality, suggesting that a sex-dependent effect was present in modulating temperature induced stress response. Consistent with the results of the room temperature study, female SNAP25^Δ3/Δ3^ mice displayed lower levels of circulating insulin in response to a glucose bolus during a GTT at thermoneutrality. The female SNAP25^Δ3/Δ3^ mice also displayed enhanced glucose tolerance compared to SNAP25^+/+^ littermates at thermoneutrality, even when lean mass was not statistically different between the two genotypes. This improved glucose handling effect is likely due to the same phenomenon displayed in our previous study that concluded male SNAP25^Δ3/Δ3^ are more sensitive to insulin irrespective of the amount of insulin secreted during a GTT (Ceddia et al., 2023). Considering that the enhanced insulin sensitivity was lost in the male SNAP25^Δ3/Δ3^ housed at thermoneutrality along with the rest of the metabolic phenotypes, this was not investigated in the female SNAP25^Δ3/Δ3^ mice as their metabolic protection phenotype persisted.

Since the female mice did not lose metabolic protection when housed at thermoneutrality as seen in the male mice, we hypothesized there might be a difference in the thermoneutral set point between our male and female mice. Thermoneutral set point refers to the temperature at which there is no longer a requirement of homeostatic body temperature regulation demanded by the environment from the organism (Škop et al., 2020). Previous studies have suggested there may be a thermoneutral set point difference between male and female mice (Kaikaew et al., 2017; Gaskill et al., 2009). However, these studies did not observe BMR in response to different environmental temperatures. Here, we show that while there may be a sex dependent difference in BMR at 21°C, there is no BMR difference between genotype nor sex between 26°C and 32°C, which extends well above the thermoneutral range for mice. Taking a closer look at Figure 3, it is important to note that as we increased temperature, male mice BMR decreased in response to the temperature increase irrespective of genotype. By contrast, female mice BMR did not respond to the rise in environmental temperature. This lack of change suggests that male mice respond to an environmental temperature shift in the 21°C–34°C range, whereas females do not. This finding is not surprising as a recent study in humans concluded that females have a lower critical temperature than males, which defines the low set point of the thermoneutral zone (Brychta et al., 2024). Repeating that study with mice may uncover a similar difference. Future studies could address this possibility.

Considering that the thermoneutral set point in our current temperature range (21°C–34°C) did not differ between sex nor genotype, our next hypothesis was that female sex hormones were responsible for the persistence of the metabolic phenotypes in SNAP25^Δ3/Δ3^ at 30°C. To investigate this, we evaluated the effect of ovariectomy on SNAP25^Δ3/Δ3^ and SNAP25^+/+^ littermates fed HFD at thermoneutrality. We observed an increase in weight and apparent loss of metabolic protection in the mice that received the OVX procedure regardless of genotype. However, the weight gain and food intake increase observed when mice underwent OVX were similar regardless of genotype, suggesting that the SNAP25^Δ3/Δ3^ and OVX mechanisms operate independently. Additionally, SNAP25^Δ3/Δ3^ mice were metabolically protected irrespective of presence of the ovaries further supporting that OVX and SNAP25^Δ3/Δ3^ mechanisms are separate. Recent literature also supports that estrogen does not have a major effect on β-AR expression levels or sensitivity (Moreira-Pais et al., 2020). Considering the significant effects seen in the SNAP25^Δ3/Δ3^ adipocytes which are downstream of the β_3_-AR, it is not surprising that norepinephrine and sex hormones are independent in our model.

The separation of mechanisms does not explain our sex-dependent phenotypes. It is well known that there are structural and genetic differences in the central nervous system between sexes due to circulating sex hormones throughout embryogenesis and later development (Sokolowski et al., 2024; Heck and Handa, 2019; Swaab et al., 2003). It is unclear how these changes manifest in sex-dependent phenotypes in temperature-dependent energy homeostasis. These developmental changes in the central nervous system may be the cause of persistent metabolic protection in the female SNAP25^Δ3/Δ3^ mice. Synaptic pruning is a key developmental process that relies on removing the synapses that do not have a strong connection during synaptic transmission responsible for many developmental phenomena such as fine motor skills and cognitive function (Kasthuri and Lichtman, 2003). Furthermore, it is plausible that increased neurotransmitter release in the synaptic cleft and sex hormone dependent effects in hypothalamic development could lead to drastic changes in temperature response, feeding behavior, and noradrenergic temperature regulation. Additional studies investigating the effect of the SNAP25^Δ3/Δ3^ mutation in an estrogen receptor knockout mouse would provide significant insight into whether estrogen-dependent structural differences in the central nervous system are responsible for the female-specific persistence of metabolic protection. Finally, using an inducible SNAP25^Δ3/Δ3^ model to repeat these studies would allow for induction of the SNAP25Δ3 mutation after development, which could indicate whether the SNAP25^Δ3/Δ3^ genotype is causing developmental differences due to the SNAP25 truncation.

Here, we have demonstrated that female SNAP25^Δ3/Δ3^ display metabolic protection phenotypes as previously demonstrated by male SNAP25^Δ3/Δ3^ mice. However, there is underlying sex dependent biology that allows female SNAP25^Δ3/Δ3^ mice to display persistent metabolic protection whereas male SNAP25^Δ3/Δ3^ mice cannot at thermoneutrality. Many previous studies have characterized the SNAP25^Δ3/Δ3^ phenotypes in male mice specifically, and future studies will further investigate the sex differences in our SNAP25^Δ3/Δ3^ mouse model.

## Materials and methods

### Animal procedures

Mice were generated through heterozygous breeding of SNAP25^+/+^ and SNAP25^Δ3/Δ3^ mice on a C57BL/6 background as described previously (Zurawski et al., 2019) and were housed at Vanderbilt University. Mice were maintained on 12-h light/12-h dark cycles with 3–5 animals per cage except during food intake monitoring, during which they were singly housed. Mice were housed at standard room temperature (∼22°C) or in thermoneutral housing conditions (∼30°C) when indicated. Mice used in this study were 8–12 weeks old of appropriate genotypes at the initiation of the study and therefore 16–20 weeks old at the end of the high fat diet challenge. Mice were maintained *ad libitum* on chow (13.5% calories from fat: 5001; LabDiet) or a HFD (60% calories from fat; 3282, Bio-Serv) when indicated. Weights and body composition measurements using the LF50 Body Composition Analyzer (Bruker) were done at the Vanderbilt Mouse Metabolic Phenotyping Center (VMMPC). Mice were euthanized by isoflurane overdose and exsanguination via cardiac puncture at the end of the study for collection of blood and tissues.

### Histology

Tissues were fixed in 10% formaldehyde overnight and subsequently stored in 70% ethanol and routinely processed. The tissues were then embedded, sectioned, and stained with H&E or were immunohistochemically stained for uncoupling protein 1 (UCP1) (ab10983, Abcam). Histological analysis was performed by the Vanderbilt Translational Pathology Shared Resource. Whole slides were imaged at ×20 magnification with a Leica SCN400 Slide Scanner by the Digital Histology Shared Resource at Vanderbilt University Medical Center.

### Histology Quantification

Adipocyte size was quantified in Fiji adapted from a method developed by Joseph Roland (https://www.vumc.org/dhsr/sites/default/files/public_files/Fiji-Adipose-Segmentation.pdf). Briefly, a TIFF image with full cell coverage from the iWAT section at a resolution of 0.5 μm per pixel was uploaded into Fiji. The image was converted to 8 bits, and the colors were inverted. The background was subtracted using the settings of Rolling Ball Radius 20.0 pixels and Sliding Paraboloid. The image was manually cropped into multiple segments to reduce file size. Morphological Segmentation with Gaussian radius 3 and Watershed Segmentation tolerance 4 was run on each segment. An image was created showing the dams from the Morphological Segmentation, and a Gaussian blur with a Sigma of 2.0 was added. The resulting image was converted to 8 bits, and the threshold settings were manually adjusted so that the cell walls were white on a black background. Analyze Particles was run with the size range of zero-infinity, producing a list of adipocyte areas in pixels. measurements below above 2,000 pixels squared were excluded. The area was converted to μm^2^. All adipocytes size measurements within this range were plotted for each individual mouse, with ∼2,000 measurements per mouse. Analyses comparing genotypes were performed using the median adipocyte size. Statistical analysis done by a two-tailed Student’s t-test with α = 0.05 using GraphPad Prism software.

### GTT

For all GTTs, mice were fasted for 5 h with *ad libitum* access to water, and fasting blood glucose, and subsequent blood glucose measurements were made from a drop of tail vein blood and analyzed using a Contour Next EZ glucometer at the indicated time points. GTTs were conducted in a 30°C room. Glucose was given by an intraperitoneal injection of 2.0 g/kg glucose.

### Hormone Analyses

Plasma for insulin during a GTT was collected from mice via tail snip and collected in Microvette CB 300 K2E tubes coated with EDTA. Blood samples were stored on ice and subsequently centrifuged at 1000 RCF 4°C for 30 min. Plasma was then isolated from the microvette tube and stored at −20°C until analysis was completed. Insulin concentrations during GTTs (Supplementary Figures S2, S4) was analyzed by radioimmunoassay at the Vanderbilt University Hormone Assay and Analytical Services Core. Data from seven mice were excluded from insulin testing during a GTT due to consistent coagulation and one suffered from cardiac arrest. Plasma for leptin analysis was collected from 8 to 12-week-old mice housed at 22°C with *ad-libitum* access to HFD which were euthanized as described above and blood collected via cardiac puncture with EDTA and again centrifuged at 1000 RCF 4°C for 30 min. Plasma was then isolated from the collection tube and stored at −20°C until analysis. Plasma for leptin was analyzed with a Luminex 100 system at the Vanderbilt University Hormone Assay and Analytical Services Core (Supplementary Figure S6).

### Energy balance

Food intake and energy expenditure were monitored in mice by the VMMPC using a Promethion system (Sable Systems International). Animals were housed over multiple days to acclimate to the facility. Body weight and composition were monitored before and after a calorimetry study.

### Study approval

The animal protocols were approved by the Vanderbilt University (Nashville) IACUCs.

## Data Availability

The raw data supporting the conclusions of this article will be made available by the authors, without undue reservation.
