# Supplementary figures and images for: Sex differences in metabolic regulation by Gi/o-coupled receptor modulation of exocytosis
Source: Front Pharmacol. 2025 Mar 19;16:1544456. doi: 10.3389/fphar.2025.1544456 (PMC11962901; doi:10.3389/fphar.2025.1544456)

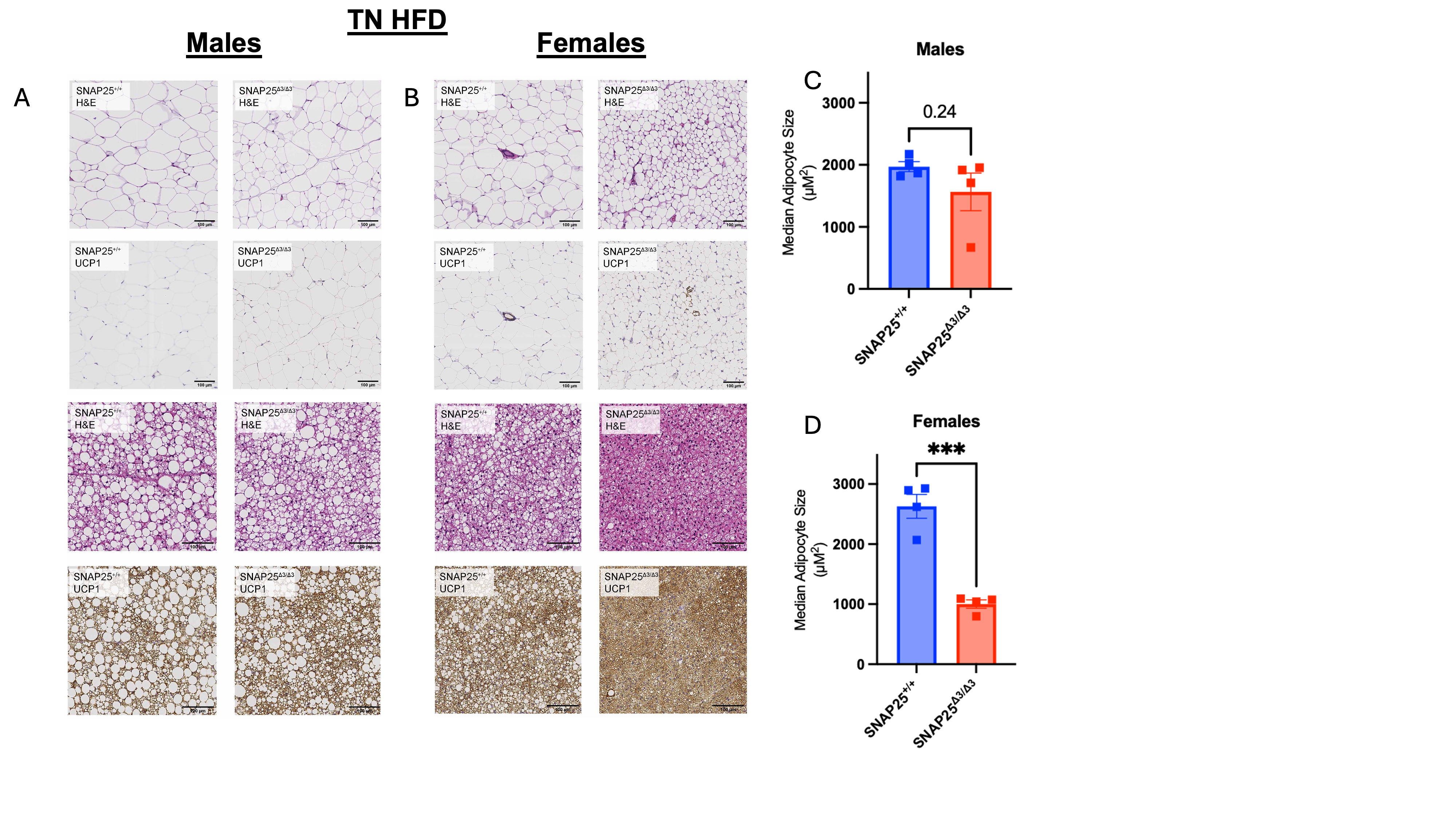

Supplement: Supplementary file 1 [file Image3.tiff]

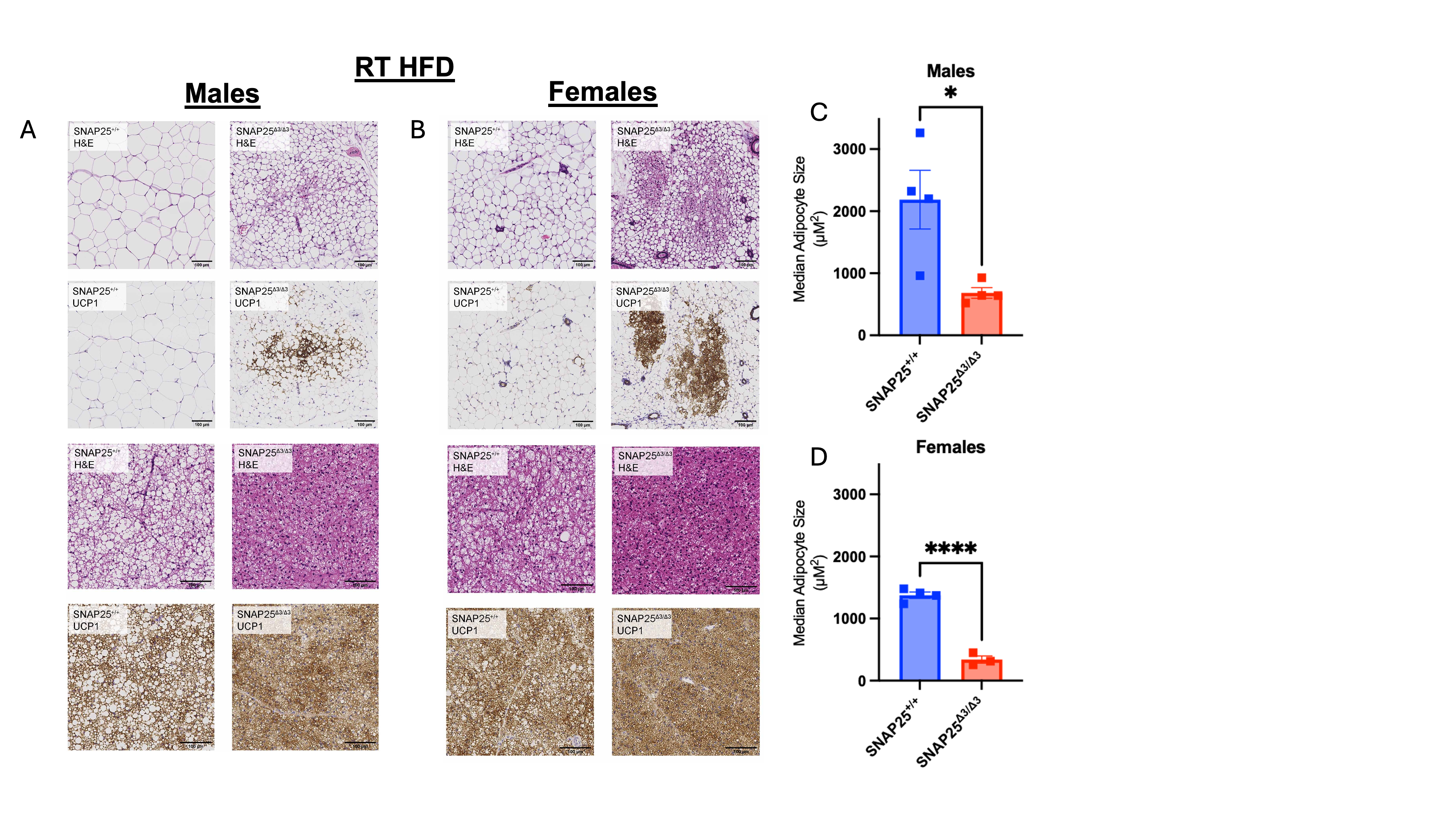

Supplement: Supplementary file 2 [file Image1.tiff]

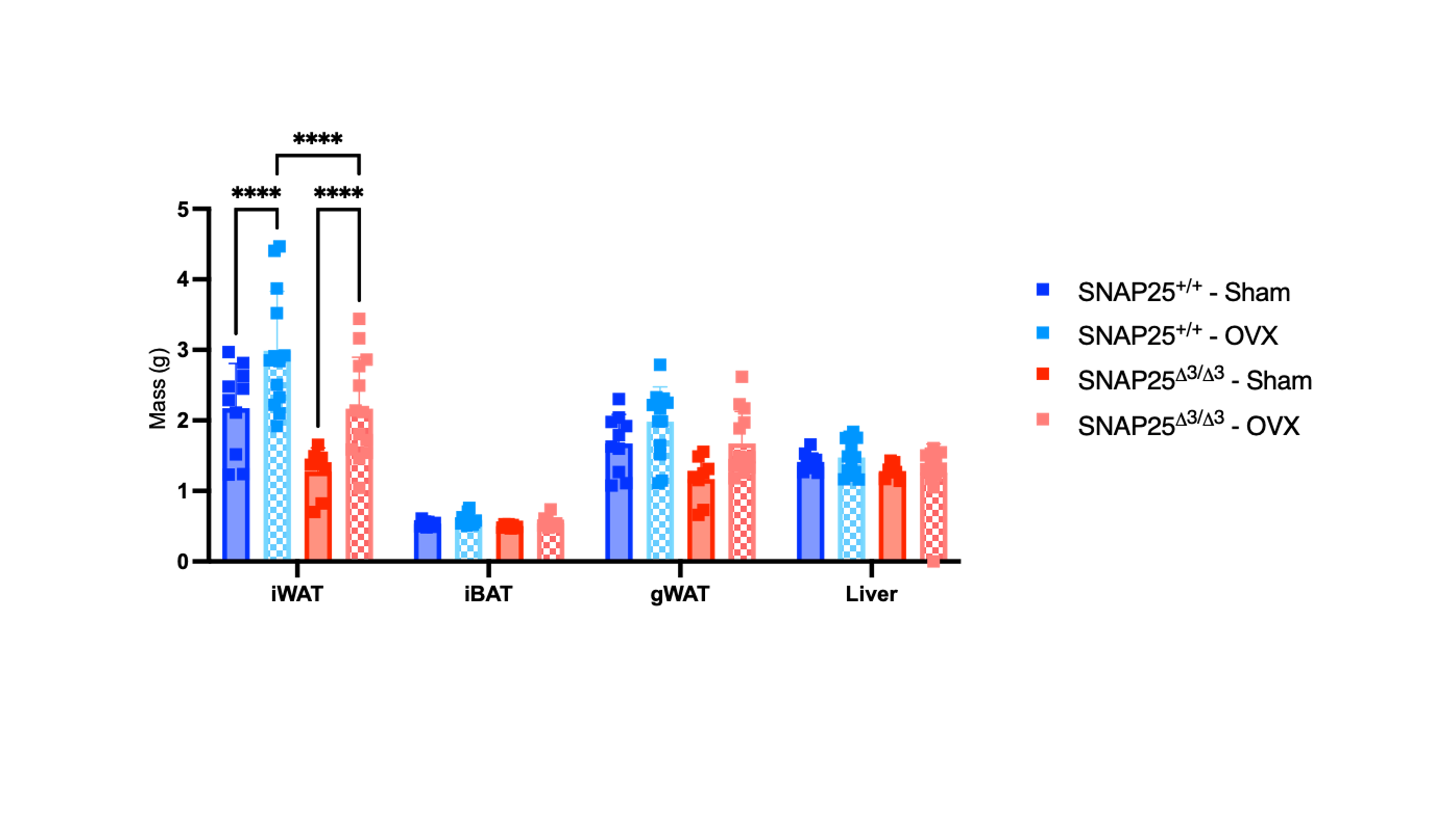

Supplement: Supplementary file 3 [file Image5.tiff]

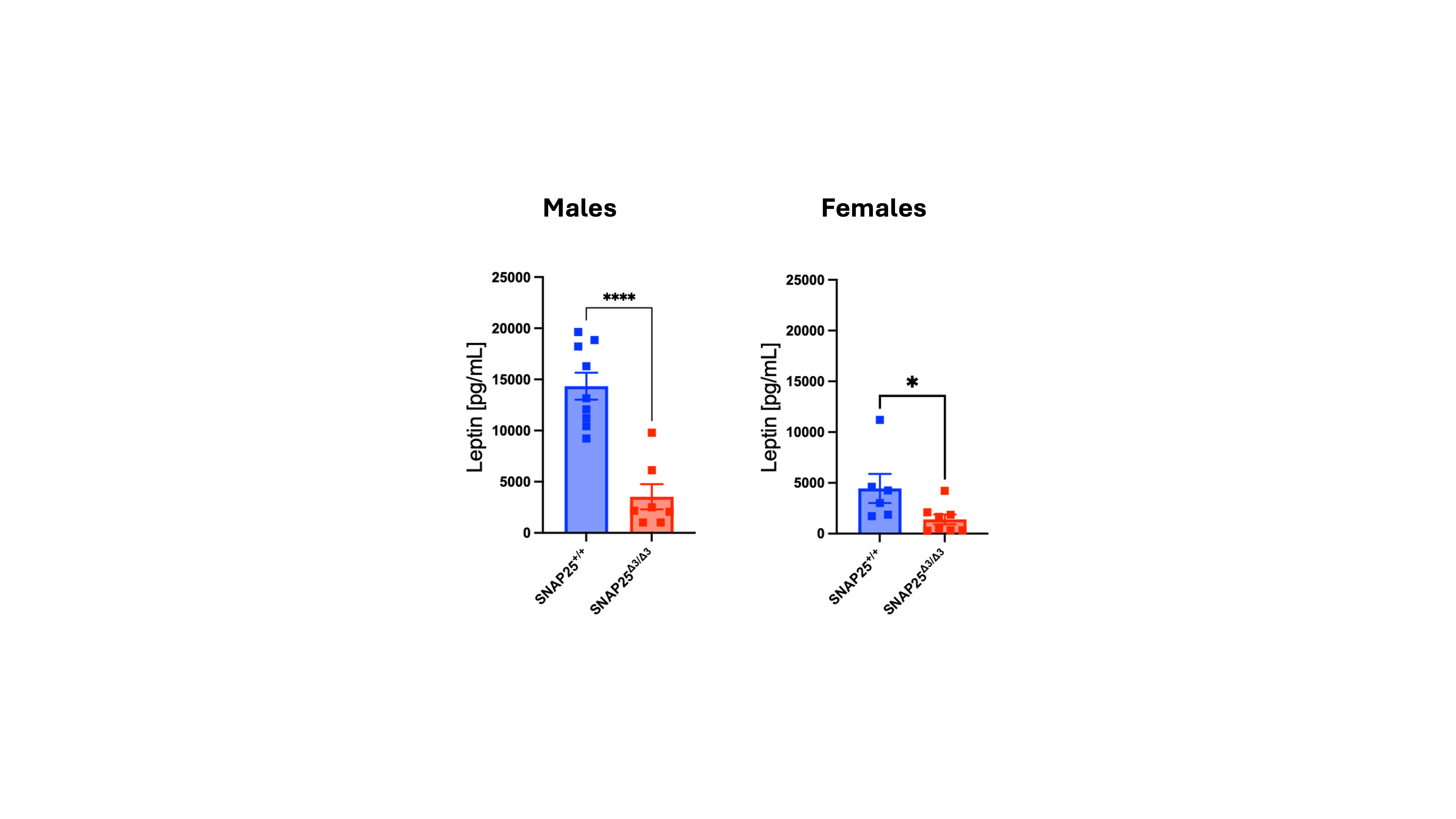

Supplement: Supplementary file 4 [file Image6.tiff]

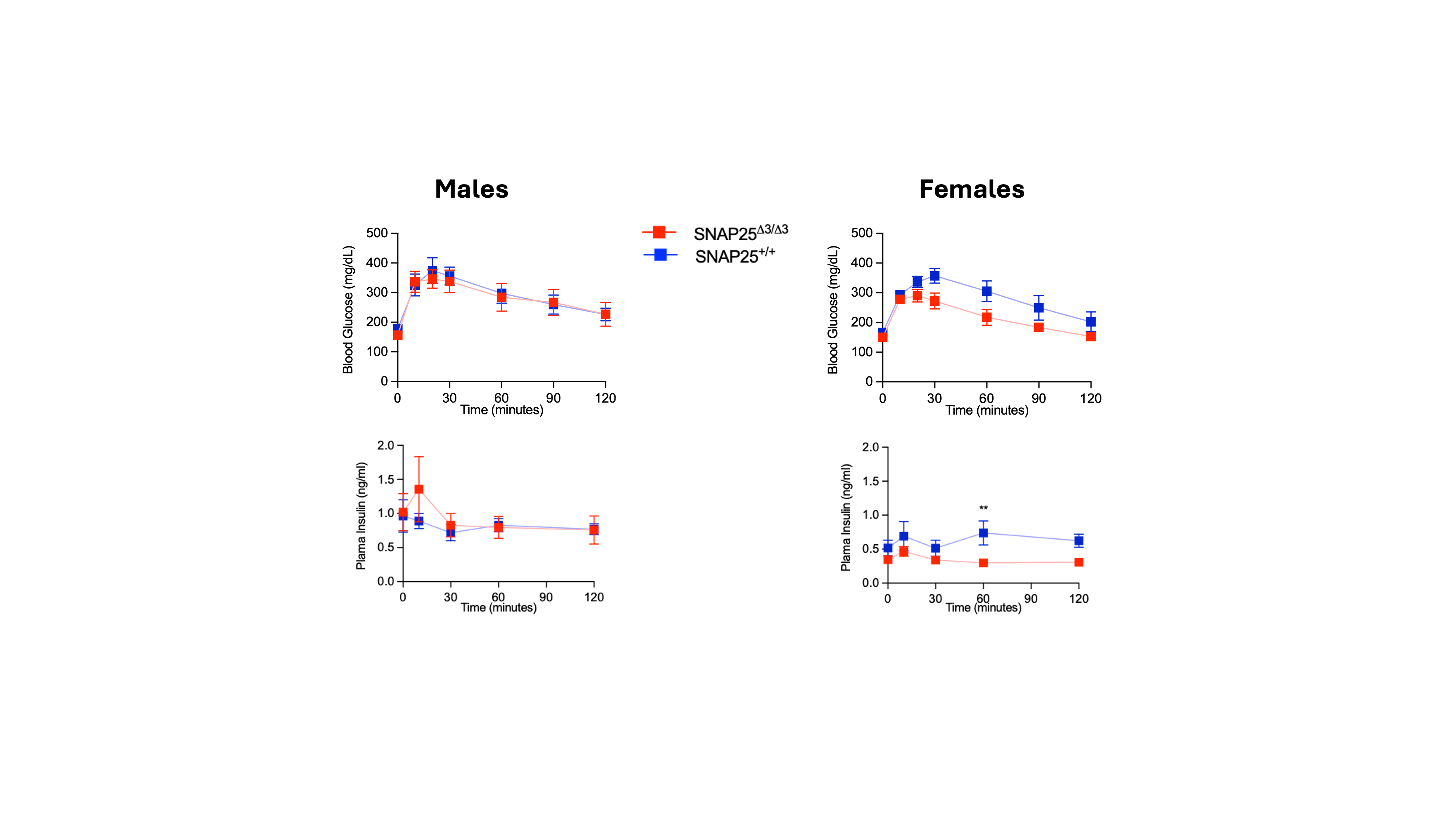

Supplement: Supplementary file 5 [file Image2.tiff]

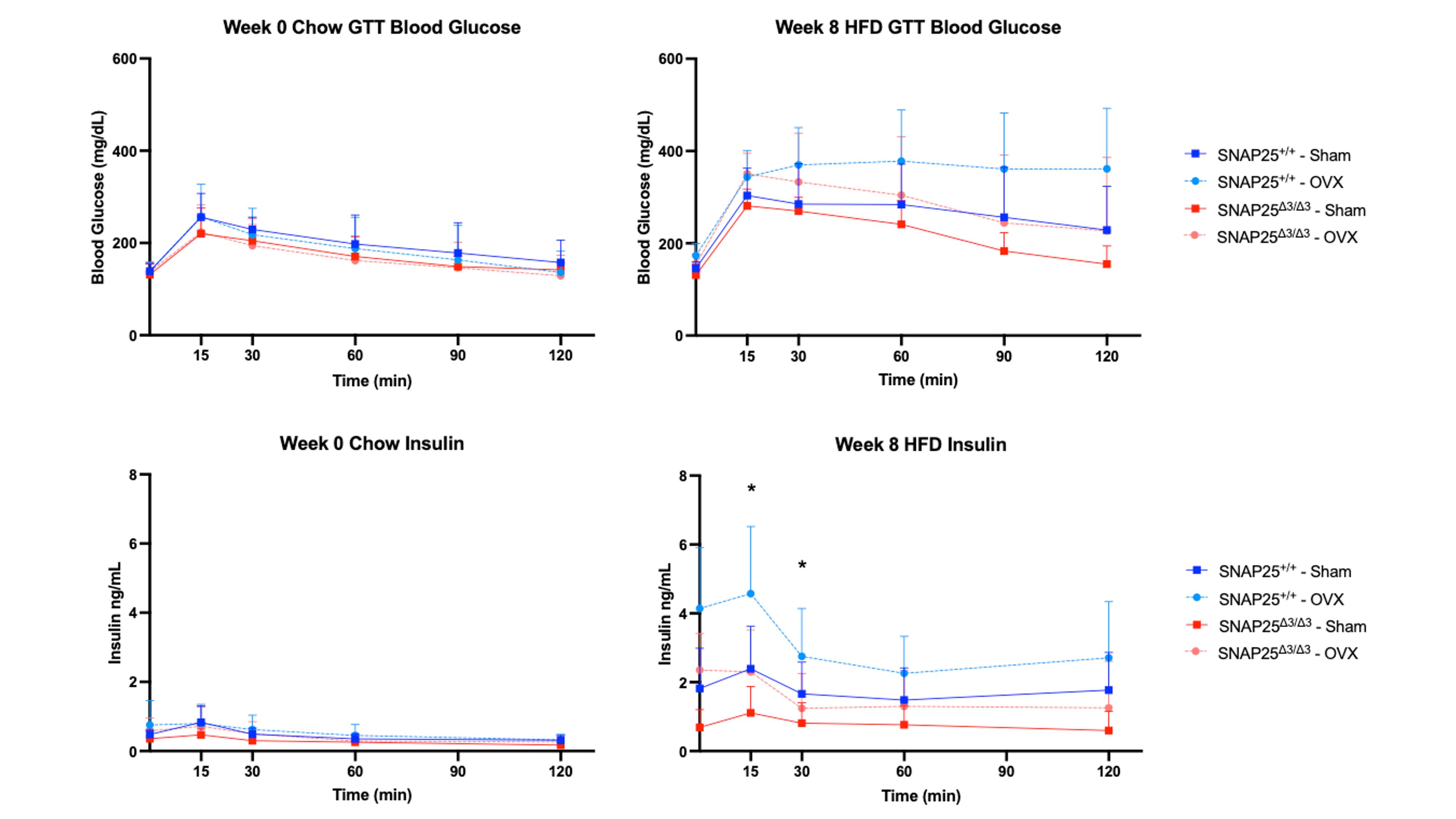

Supplement: Supplementary file 6 [file Image4.tiff]

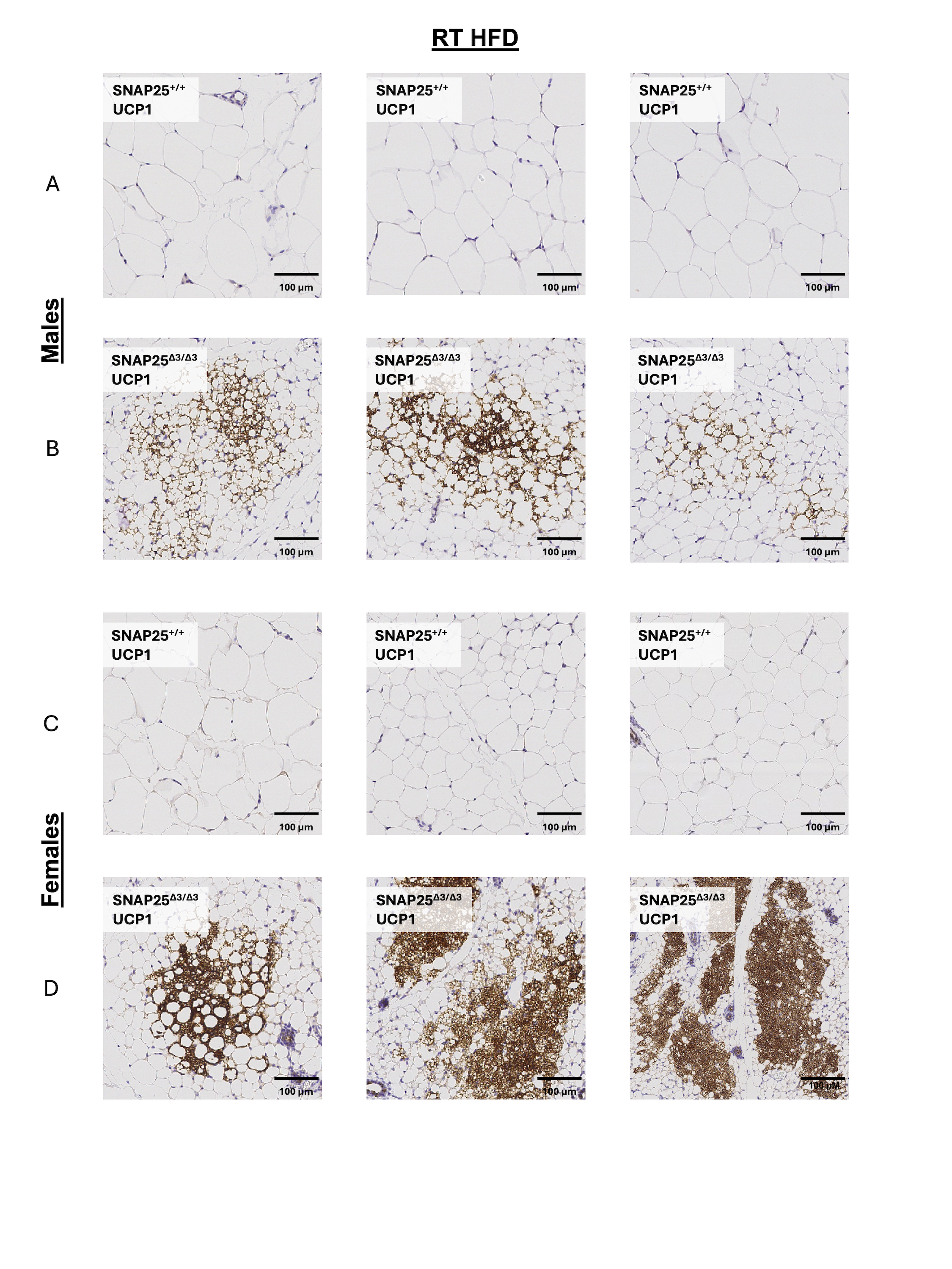

Supplement: Supplementary file 7 [file Image7.tiff]
